# Targeting hyperactivated DNA-PKcs by KU0060648 inhibits glioma progression and enhances temozolomide therapy via suppression of AKT signaling

**DOI:** 10.18632/oncotarget.10864

**Published:** 2016-07-27

**Authors:** Tian Lan, Zitong Zhao, Yanming Qu, Mingshan Zhang, Haoran Wang, Zhihua Zhang, Wei Zhou, Xinyi Fan, Chunjiang Yu, Qimin Zhan, Yongmei Song

**Affiliations:** ^1^ State Key Laboratory of Molecular Oncology, Cancer Institute and Hospital, Chinese Academy of Medical Sciences and Peking Union Medical College, Beijing, China; ^2^ Department of Neurosurgery, Sanbo Brain Hospital, Capital Medical University, Beijing, China

**Keywords:** p-DNA-PKcs, glioma, KU0060648, AKT, temozolomide

## Abstract

The overall survival remains undesirable in clinical glioma treatment. Inhibition of DNA-PKcs activity by its inhibitors suppresses tumor growth and enhances chemosensitivity of several tumors to chemotherapy. However, whether DNA-PKcs could be a potential target in glioma therapy remains unknown. In this study, we reported that the hyperactivated DNA-PKcs was profoundly correlated with glioma malignancy and observe a significant association between DNA-PKcs activation and survival of the glioma patients. Our data also found that inhibition of DNA-PKcs by its inhibitor KU0060648 sensitized glioma cells to TMZ *in vitro*. Specifically, we demonstrated that KU0060648 interrupted the formation of DNA-PKcs/AKT complex, leading to suppression of AKT signaling and resultantly enhanced TMZ efficacy. Combination of KU0060648 and TMZ substantially inhibited downstream effectors of AKT. The *in vivo* results were similar to those obtained *in vitro*. In conclusion, this study indicated that inhibition of DNA-PKcs activity could suppress glioma malignancies and increase TMZ efficacy, which was mainly through regulation of the of AKT signaling. Therefore, DNA-PKcs/AKT axis may be a promising target for improving current glioma therapy.

## INTRODUCTION

Characterized by a median survival ranging from 5 to 59 months and accounting for 70% of adult primary central nervous system tumors, glioma has been regarded as the most deadly brain malignancy that is essentially incurable [1, 2]. Currently, TMZ is widely used in glioma treatment and has been proved to benefits patients [3, 4]. Unfortunately, drug resistance restricts the clinical application of TMZ and therefore contributes to the dismal outcome of glioma patient [5, 6]. This challenging problem attributes to multitudes of aberrant molecular changes, such as MGMT promoter methylation, α5β1 integrin expression, sonic hedgehog and notch pathway activation [7–10]. However, none of them has been successfully translated into clinical application and resultantly improved TMZ efficacy [11–14]. Thus, comprehensive understanding of TMZ resistance in glioma is urgently needed.

DNA-PKcs is a nuclear protein serine/threonine kinase which plays a pivotal role in the non-homologous end-joining (NHEJ) pathway for DNA double-strand break (DSBs) repair [15]. Recently, series of studies have characterized that DNA-PKcs can also act as molecular promoter to control a wide array of cellular functions, such as cell cycle, metabolism, hypoxia, inflammatory response [16–19]. Activation of DNA-PKcs regulates various tumor-promoting molecules, such as maintaining the stability of Snail1, Chk1–Claspin complex, or enhancing the transcriptional activity of p53 and androgen receptor (AR), and resultantly mediates the progression of colon cancer, prostate adenocarcinoma, hepatocellular carcinomas (HCC) and other human cancers [20–23]. However, the role of DNA-PKcs in glioma progression remains to be elucidated.

As a result of its functional diversity, DNA-PKcs has been documented to play a critical role in the development of chemoresistance. Ganesh R. Panta *et al* noted that DNA-PKcs activated prosurvival NF-κB pathway through MEK/ERK signaling and opposed the apoptotic response following chemotherapeutic agent [24]. Furthermore, Suk-Bin Seo *et al* reported that TRAIL inhibited the activation of DNA-PKcs/AKT/GSK-3β pathway and thereby sensitized tumor cells to vinblastine and doxorubicin [25]. Thus, exploring the biologic function of DNA-PKcs and its mediated signaling may provide an ideal therapeutic target for overcoming TMZ resistance in glioma treatment.

In this study, we surveyed the p-DNA-PKcs (Ser 2056) level in human glioma samples and observed that hyperactivation of DNA-PKcs was closely associated with both malignant progression and poor clinical outcome of glioma patients. We further explored the potential correlation between inhibition of DNA-PKcs and TMZ efficacy in glioma. The results demonstrate a striking synergistic effect between DNA-PKcs inhibitor KU0060648 and TMZ in glioma cells. Inhibition of DNA-PKcs enhances TMZ sensitivity mainly via suppression of AKT activation. This study provides a potential target for evaluating glioma progression and improving TMZ efficacy in glioma therapy.

## RESULTS

### p-DNA-PKcs expression positively correlates with poor prognosis of patients with glioma

**Figure 1:**
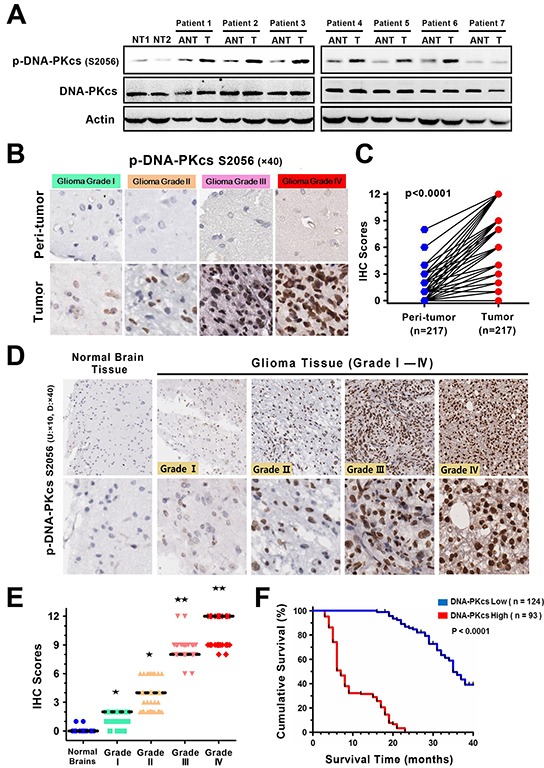
p-DNA-PKcs expression associates with tumor progression and poor prognosis of gliomas **A.** Immunoblotting analysis of p-DNA-PKcs (S2056) expression in 2 normal human brains from trauma, 7 paired primary glioma tissues (T) and matched adjacent nontumorous tissues (ANT) from the same patient (Patients No.1,2: WHO grade IV; No.3,4: WHO grade III; No.5,6: WHO grade II; No.7: WHO grade I). Actin was used as a loading control. **B, C.** Immunohistochemistry (IHC) study on p-DNA-PKcs expressions between gliomas and paired normal tissues. Representative IHC images (B) (magnification, ×40 as indicated) and statistical analysis (C) (*p* < 0.001, *t* test). **D.** IHC staining of p-DNA-PKcs in different grades of gliomas and normal brain tissues (magnification, ×10 and ×40 as indicated). **E.** Correlation between p-DNA-PKcs expression and tumor grade in surveyed cohort. (Bars, median expression values of IHC scores; ★, *p* < 0.05; ★★, *p* < 0.001; Wilcoxon rank sum test). **F.** Kaplan-Meier curves of glioma patients with low vs. high level of p-DNA-PKcs (n=217; *p* < 0.0001, log-rank test).

To investigate the activated status of DNA-PKcs in glioma progression, we first evaluated the expression levels of phosphorylated-DNA-PKcs (Ser 2056, p-DNA-PKcs S2056) in human gliomas and their paired adjacent nontumorous tissues or normal human brain tissues using immunoblotting. As shown in Figure 1A, p-DNA-PKcs was significantly higher in 7 human glioma specimens than their respective adjacent nontumorous tissues or 2 normal brains. Immunohistochemistry (IHC) analysis in a cohort of 217 paraffin-embedded glioma samples further confirmed the overexpression of p-DNA-PKcs in 57.2% of gliomas (124/217) as compared with corresponding non-tumor tissues (62/217, 28.6%; Figure 1B - 1C, Supplementary Table S1). We then assessed the relationship between p-DNA-PKcs levels and the clinical features of glioma. Strong expressions of p-DNA-PKcs were positively correlated with higher grade tumor status (Figure 1D - 1E, Supplementary Figure S1), and also closely associated with worse survival of glioma as determined by the Kaplan-Meier and log-rank tests for survival analysis (OS, p < 0.0001; Figure 1F). More importantly, multi-variate analysis through Cox regression model with all 6 parameters (p-DNA-PKcs level, age, gender, tumor location, debulking degree, tumor grade) identified the independent prognostic significance of p-DNA-PKcs (hazard ratio: 3.052; p < 0.001; 95% CI: 2.204 - 4.572), which was not linked to known prognostic factors such as ages and tumor grades (Supplementary Table S2).

Aiming to comprehend if the activated DNA-PKcs arose from DSBs, we selected 155 patients with primary glioma occurrence and null chemo- or radiotherapy before surgery from our glioma cohort, then surveyed the expression of γH2AX. On the contrary to that p-DNA-PKcs levels were positively associated with glioma grades, γH2AX did not appear to be discriminatingly expressed among different grades of glioma tissues (Supplementary Figure S2A). Further analysis certified that there was not correlation between expression of γH2AX and p-DNA-PKcs, suggesting that activation of DNA-PKcs in glioma was not exclusively in response to DSBs (Supplementary Figure S2B). Taken together, these results indicated that p-DNA-PKcs expression was abnormally overexpressed in gliomas and dysregulated expression of p-DNA-PKcs correlated with malignant development and poor prognosis in clinical glioma patients.

### Inhibition of DNA-PKcs activity reduces glioma growth and sensitizes cells to TMZ

**Figure 2:**
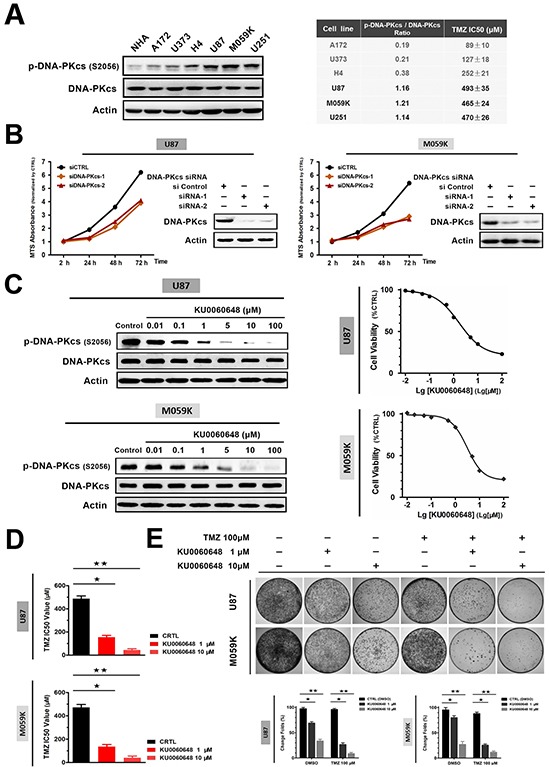
Inhibition of DNA-PKcs activation suppresses cell proliferation and sensitizes glioma to TMZ in vitro **A.** Assessment of p-DNA-PKcs/DNA-PKcs expression in human glioma cell lines by immunoblotting. Temozolomide (TMZ) IC_50_ values were presented in right table. **B.** siRNA-mediated knockdown of DNA-PKcs reduced glioma cell lines U87 and M059K proliferation. Immunoblotting confirmed the knockdown efficacy, and cell growth curve was measured by MTS assay. **C.** Pharmacologic characteristic of KU0060648 on p-DNA-PKcs inhibition following 6-hour incubation in U87 and M059K cells (left). Proliferations of two cell lines were investigated by MTS assay after 72-hours treatment (right). **D.** TMZ IC_50_ of U87 and M059K in the absence or presence of KU0060648. Two cell lines were incubated for 3 days in a range of concentrations of TMZ with or without of 1 or 10 μM KU0060648 and IC_50_ value was then calculated by MTS assay (Bars, SD; ★, *p* < 0.05; ★★, *p* < 0.001, one-way ANOVA test). **E.** Colony formation assay evaluating the growth of U87 and M059K cells in treatment of TMZ (100 μM) and KU0060648 (1 or 10 μM) either alone or in combination for 14 days. Cells were stained with 0.1% crystal violet (Bars, SD; ★, *p* < 0.05; ★★, *p* < 0.001; one-way ANOVA test).

Next we sought to address the expression of p-DNA-PKcs in glioma cell lines. Data in Figure 2A revealed that, in contrast to normal human astrocyte (NHA) which possessed an undetectable level of activated DNA-PKcs, p-DNA-PKcs were expressed in a panel of glioma cells. We also examined the γH2AX level in these cells. However, none of them demonstrated an obvious band of γH2AX (Supplementary Figure S3). Notably, cell lines with high levels of p-DNA-PKcs (U87 and M059K) demonstrated higher IC_50_ values of TMZ compared with A172 and H4. We then determined whether DNA-PKcs contributed to glioma cell growth and TMZ sensitivity. Strikingly, DNA-PKcs siRNA transfection effectively suppressed the proliferation of U87 and M059K cells and enhanced the cytotoxic effect of TMZ using MTS assays (Figure 2B and Supplementary Figure S4).

We further characterized if inhibition of DNA-PKcs could suppress glioma cell growth or enhance TMZ sensitivity using KU0060648, a novel DNA-PKcs inhibitor which was applied in previous researches [26, 27]. As shown in Figure 2C, KU0060648 inhibited the activation of DNA-PKcs (left panel of Figure 2C) and the proliferation of glioma cells (right panel of Figure 2C) in a dose-dependent manner. Moreover, KU0060648-mediated DNA-PKcs inhibition led to sensitization of TMZ in glioma cells. Addition of 1 or 10 μM of KU0060648 increased TMZ efficacy remarkably with the IC_50_ values dropping from 487 ± 24 μM to 154 ± 16 μM or 44 ± 12 μM, respectively in U87 (Figure 2D). Similar results were observed in M059K (Figure 2D). To better understand the effect of KU0060648 on TMZ sensitivity, glioma cell lines with lower levels of p-DNA-PKcs, namely H4 and U373, were also investigated. In contrast to U87 and M059K, KU0060648 only mildly restored the sensitivity to TMZ in H4 and U373 (Supplementary Figure S5), which supported the idea that KU0060648-sensitized TMZ depended on the suppression of p-DNA-PKcs levels.

We subsequently assessed the long-term influence of KU0060648 on tumor growth and TMZ sensitivity using anchorage-dependent colony formation assay. Figure 2E elucidated that co-administration of KU0060648 and TMZ substantially reduced colony formation compared with each single agent. Collectively, our data revealed that inhibition of DNA-PKcs by KU0060648 could suppress proliferation and enhance the TMZ cytotoxicity in glioma cells.

### KU0060648 enhances TMZ-induced apoptosis of glioma cells

**Figure 3:**
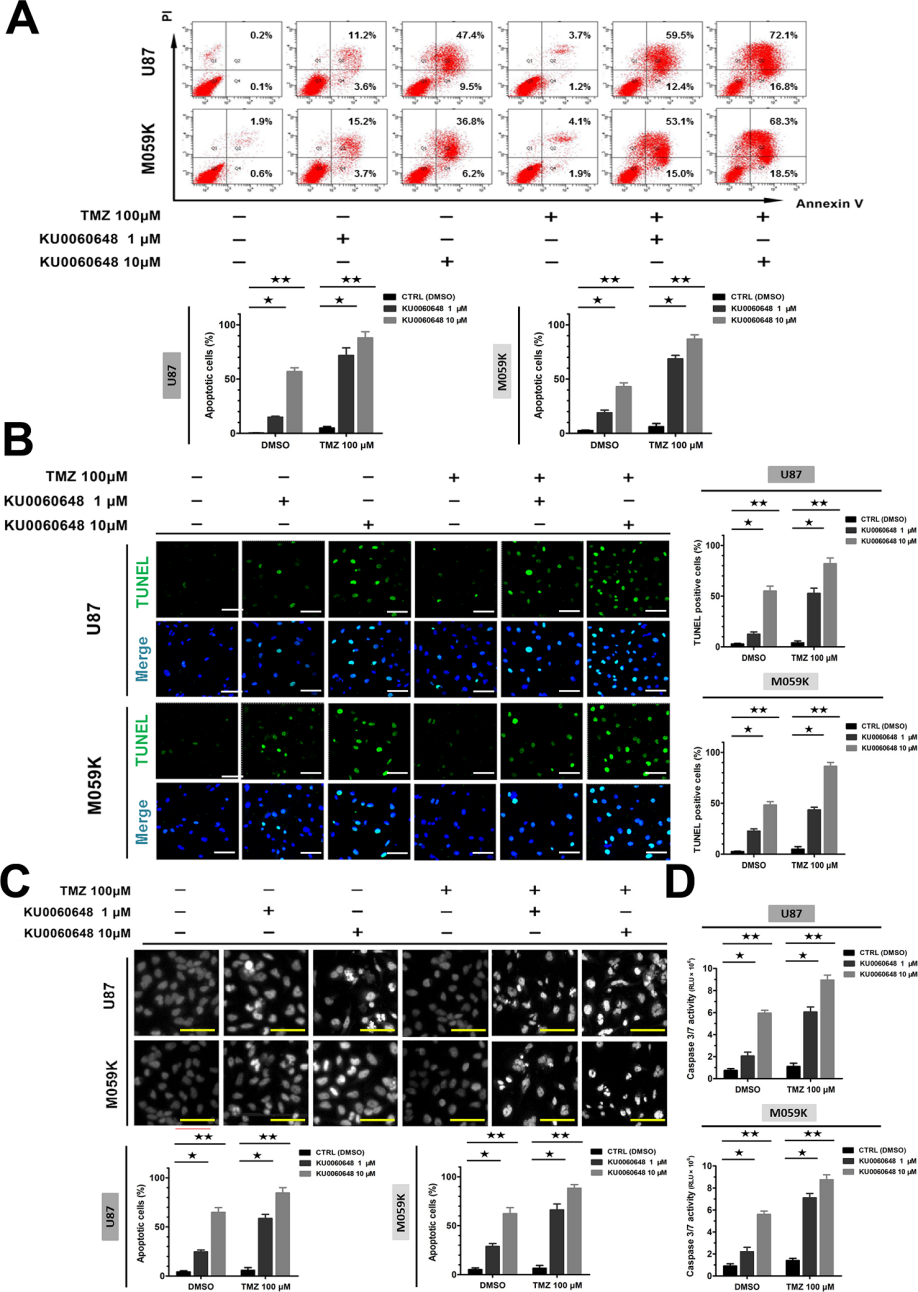
Effects of combination treatment of KU0060648 and TMZ on the apoptosis of glioma cells U87 (up) and M059K (down) cells were treated with 100 μM TMZ, 1 or 10 μM KU0060648, and their combinations for 48-hours, cell apoptosis was evaluated by Annexin V/PI FCM assay **A.** TUNEL assay **B.** and Hoechst 33258 staining **C.** Caspase-3/7 activity was detected in the above treatments **D.** (Scale bars: 100 μm; bars, SD; ★, *p* < 0.05; ★★, *p* < 0.001; one-way ANOVA test).

To investigate the effect of KU0060648, TMZ or KU0060648/TMZ on apoptosis of glioma cells, U87 cells were treated with these agents alone or their combination for 48 hours and then cell apoptosis was assessed by flow cytometry (FCM). As shown in Figure 3A, 1 μM or 10 μM KU0060648 alone was able to increase the apoptotic rates to 14.8% and 56.9% respectively in U87 cells. 100 μM TMZ exerted minimal effect, while KU0060648 dose-dependently enhanced the apoptotic rate of TMZ treatment, which was certified in TUNEL assay (Figure 3B). To further classify the proapoptotic effect of combination treatment, morphological changes of U87 cells were evaluated using Hoechst staining (Figure 3C). 100 μM TMZ alone did not induce evident morphological alternations in U87 cells. 1 or 10 μM KU0060648 alone, or in combination with 100 μM TMZ could arouse the distinct morphology features of apoptosis, including the formation of apoptotic body and early coalescence of nuclear chromatin, margination and nuclear shrinkage. Similar results were obtained in M059K (Figure 3A - 3C). Notably, we discovered that treatment of KU0060648 increased the caspase 3/7 activity-promoting effect of TMZ in both U87 and M059K (Figure 3D), suggesting the synergistic augment of apoptosis caused by KU0060648 and TMZ majorly depended on the activation of caspases.

In order to determine whether KU0060648 inhibited glioma growth and sensitized temozolomide through suppression of DNA damage repair, we detected γH2AX foci, a frequently used assay visualizing the occurrence of DSBs, in KU0060648 single agent group or co-administrated with TMZ (Supplementary Figure S6). Indeed, there was undetectable level of background foci formation per cell in DMSO-treated control group, which was not increased by sole KU0060648 (data not shown). Such a finding prompted that KU0060648 exerted its anti-malignancy effects on glioma cells via a DNA damage repair independent manner, which consisted with the effect of NU7441, another well-known DNA-PKcs specific inhibitor, in a previous study [28]. Exposure of 100 μM temozolomide alone did form γH2AX foci in both U87 and M059K cells. However, it declined rapidly, as only 11% (U87) and 16% (M059K) remained respectively after 120 hours. Additional KU0060648 did not affect the level of TMZ-induced foci formation but significantly retarded the loss of γH2AX foci. On the basis of these results, we postulated that suppressing glioma malignancies and enhancing TMZ efficacy by DNA-PKcs inhibitor might not merely depend on inhibition of DSBs repair, and thus, DNA-PKcs inhibitors can be used as single or synergized with TMZ against glioma.

### Additional KU0060648 profoundly suppresses glioma cell invasion and angiogenesis

**Figure 4:**
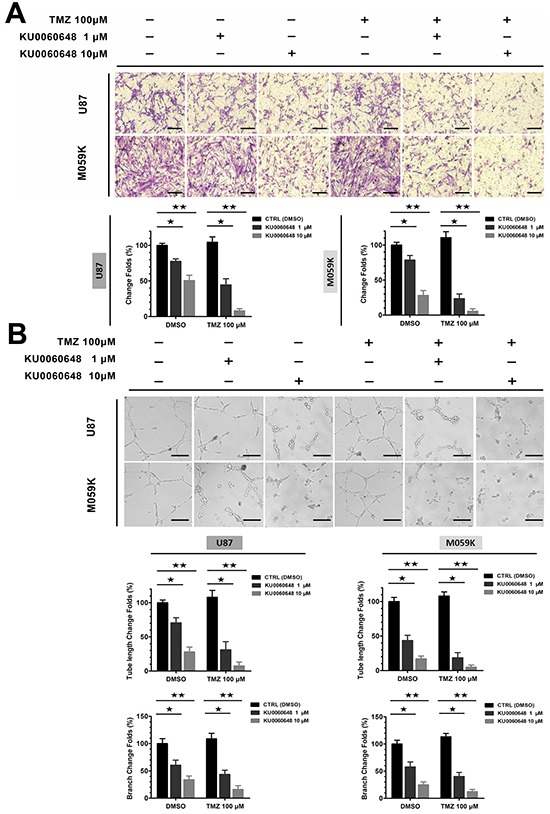
Effects of combination treatment of KU0060648 and TMZ on the invasion and angiogenesis of glioma cells **A.** Cell invasion was quantified by Matrigel coated Transwell assay in U87 and M059K cells with 100 μM TMZ, 1 or 10 μM KU0060648, and their combinations treatment. Penetrated cell were counted using NIH ImageJ (Scale bars: 100 μm; bars, SD; ★, *p* < 0.05; ★★, *p* < 0.001; one-way ANOVA test). **B.** Treatment of TMZ (100 μM) and KU0060648 (1 or 10 μM) either alone or in combination inhibited glioma angiogenesis. Tube length and branch were quantified using NIH ImageJ (Scale bars: 100 μm; bars, SD; ★, *p* < 0.05; ★★, *p* < 0.001; one-way ANOVA test).

Highly invasive and angiogenic nature of glioma contributes to the dismal outcome. However, instead of fighting against those aggressive characteristics, TMZ efficacy could be compromised by glioma invasion and angiogenesis. To determine whether KU0060648 inhibited invasive properties of glioma, U87 cells were treated with KU0060648 alone or in combination with TMZ for 16 hours and then assessed by Matrigel-coated Transwell assay. As shown in Figure 4A, 100 μM TMZ could not inhibit the invasiveness of U87, but rather slightly increased cells invasion. In contrast, 1 or 10 μM KU0060648 alone significantly decreased the invasive ability of U87 cells and profoundly prompted the anti-invasive ability of TMZ.

Furthermore, HUVEC tube formation assay indicated that schedule of KU0060648 treatment dose-dependently inhibited *in vitro* angiogenesis. 1 or 10 μM KU0060648 alone reduced the tube formation index of HUVECs, and dose-dependently enhanced the anti-angiogenic ability of 100 μM TMZ (Figure 4B) which was ineffective while promoted angiogenesis as single agent in U87 cells. Similar results were also obtained in M059K cells (Figure 4A and 4B).

### KU0060648 represses glioma growth mainly via inhibition of DNA-PKcs/AKT axis

**Figure 5:**
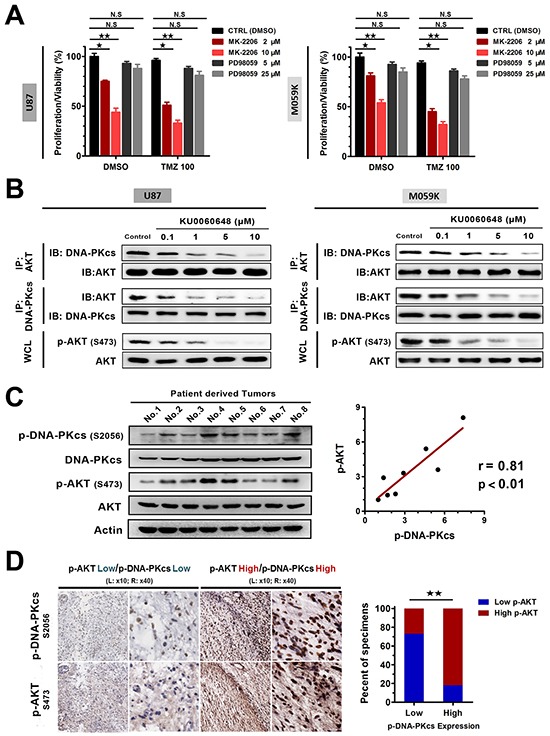
KU0060648 inhibits glioma proliferation and enhances the cytotoxic effect of TMZ in glioma cells through AKT inhibition **A.** Cell proliferation/viability of U87 and M059K was determined by MTS assay after 3-day incubation with 2 or 10 μM MK2206, 5 or 25 μM PD98059, or in combination with 100 μM TMZ. (Bars, SD; ★, *p* < 0.05; ★★, *p* < 0.001; N.S, no significance; one-way ANOVA test). **B.** KU0060648 dose response (6 hours) in disrupting DNA-PKcs/AKT interaction. Whole cell lysates (WCL) were immunoprecipitated with the antibodies against the indicated proteins. Immunocomplexes were then immunoblotted using antibodies against the indicated proteins, which is accompanied with p-AKT (S473) expression. **C.** Immunoblotting (left) and correlation analyses (right) of p-DNA-PKcs expression with the levels of p-AKT in 8 freshly collected human glioma samples (Pearson's correlation coefficients). Actin was used as loading controls. **D.** The expression levels of p-AKT were associated with the expression of p-DNA-PKcs in 42 primary human glioma specimens, which was quantified by IHC. Two representative cases with serial sections staining were shown (left) (magnification, ×10 and ×40 as indicated). Percentage of samples showed low or high p-AKT expression relative to the levels of p-DNA-PKcs (right). (★★, *p* < 0.001; chi-square test).

To dissect the potential mechanism by which DNA-PKcs inhibition mediated antiproliferative effect and synergized with TMZ, we treated U87 and M059K cells with AKT inhibitor MK-2206 or MEK inhibitor PD98059 either alone or in combination with TMZ. After 3-day administration, both single and combination strategies of MK-2206 treatment resulted in a substantial decrease in cell viability. However, PD98059 did not induce additional growth inhibition in glioma cells, suggesting that AKT signaling contributed to the biological effect of KU0060648 (Figure 5A). We further observed that KU0060648 caused a strong and dose-dependent decrease in AKT phosphorylation (p-AKT Ser 473) of U87 and M059K cells using immunoblotting (Figure 5B), which indicated that inhibition of DNA-PKcs could suppress the activation of AKT in glioma cells.

To exclude the off-target effect of KU0060648, especially its nature of ATP competitive inhibitor which can suppress the AKT activity, we investigated the inhibition of DNA-PKcs-AKT axis by KU0060648 after depleting DNA-PKcs in U87 and M059K (Supplementary Figure S7). RNAi specifically repressed the expression of DNA-PKcs and the activity of AKT. After knockdown of the DNA-PKcs, KU0060648 did not exert additional inhibitory effect on AKT phosphorylation. These findings indicated that KU0060648 was a specific inhibitor of DNA-PKcs rather than PI3K-AKT signaling, which was consistent with a previous study [26].

We then performed immunoprecipitation to evaluate if dephosphorylation of DNA-PKcs by KU006048 led to an immediate dissociation of AKT from DNA-PKcs. As expected, KU0060648 disrupted the formation of DNA-PKcs/ AKT complex in a dose dependent manner and correspondingly inhibited the activation of AKT (Figure 5B).

Especially, analyses of 8 freshly collected glioma specimens clarified a positively clinical relevance between p-DNA-PKcs expression and p-AKT (Figure 5C), which was confirmed by a validate cohort of 42 glioma patients with IHC staining (Figure 5D). These data further unearthed a functional link between p-DNA-PKcs and p-AKT in glioma patients, supporting the notion that DNA-PKcs contributed to glioma aggressiveness via activating AKT signaling.

### Combination of KU0060648 and TMZ inhibits downstream effectors of AKT and regulates malignancies-related molecules

**Figure 6:**
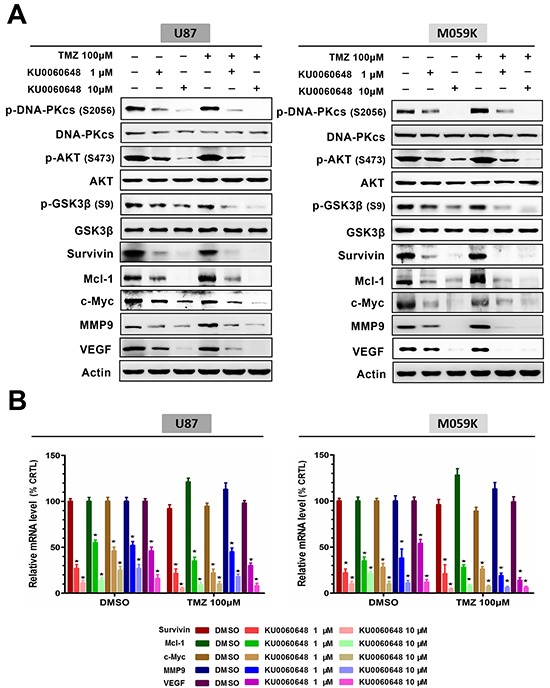
Combination of KU0060648 and TMZ inhibits the activation of DNA-PKcs/AKT axis and regulates malignancy-related effectors expression **A.** Immunoblotting analysis of p-AKT (S473), p-GSK3β (S9), survivin, Mcl-1, c-Myc, MMP9 and VEGF expressions in U87 and M059K cells treated with TMZ (100 μM) and KU0060648 (1 or 10 μM) either alone or in combination for 24 hours. **B.** Real time PCR examined the mRNA level of survivin, Mcl-1, c-Myc, MMP9 and VEGF in the treated U87 and M059K cells. (Bars, SD; ★, *p* < 0.05; one-way ANOVA test).

To identify which downstream effectors of AKT signaling were involved in mediating KU0060648 enhanced TMZ efficacy, we extracted total proteins from U87 and M059K cells after treatment with control solvent, 1 or 10 μM KU0060648, 100 μM TMZ or their combination. KU0060648 dose-dependently inhibited AKT signaling downstream effectors, such as pro-proliferative factor c-Myc, anti-apoptotic proteins survivin or Mcl-1, invasion related molecule MMP-9 and angiogenesis promoting factor VEGF (Figure 6A). Besides, activity of GSK-3β, the acknowledged direct substrates of AKT, was also suppressed by KU0060648 treatment (Figure 6A). It was noteworthy that TMZ alone did increase, although not enormously, the activities of DNA-PKcs and its downstream AKT, leading to elevate expression of pro-invasion and pro-angiogenesis effectors. Significantly, combination of KU0060648 enhanced the inhibitory effect on the DNA-PKcs/AKT signaling compared with each agent alone (Figure 6A), which suggested an underlying machinery of synergy between KU0060648 and TMZ. Similar trends were also obtained in mRNA level using qPCR (Figure 6B).

### KU0060648 synergizes with TMZ to inhibit the growth of glioma tumor *in vivo*

**Figure 7:**
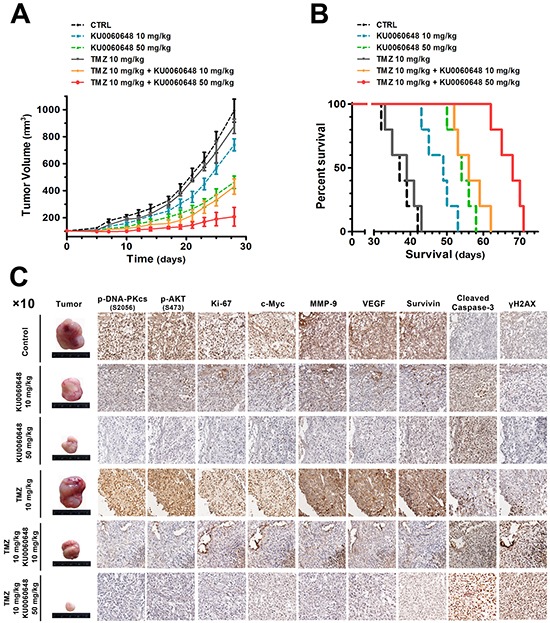
Antitumor activity of KU0060648 and in combination with TMZ against U87 xenografts **A.** Tumor volume (expressed as the mean ± SEM) of U87-beared mice in treatment of KU0060648 (10 or 50 mg/kg, once daily, i.p.) and TMZ (10 mg/kg, thrice a week, i.p.) alone, or co-administrated KU0060648 (10 or 50 mg/kg, once daily, i.p.) and TMZ (10 mg/kg, thrice a week, i.p.). Tumor size was measured every three days for the indicated period starting at day 14. The observation period was 30 days. Control mice were received the 0.9% saline. n=5 per group. **B.** Kaplan-Meier curves for illustration of the survival periods of xenograft-bearing mice in the treatment. n=5 per group **C.** IHC staining of subcutaneous xenografts samples from each treatment group. Representative IHC staining of p-DNA-PKcs (S2056), p-AKT (S473), Ki-67, c-Myc, MMP-9, VEGF, Survivin, cleaved caspase-3 and γH2AX in serial sections (magnification, ×10).

On the basis of the *in vitro* data, it was pivotal to determine if KU0060648 could synergistically promote the antitumor effect of TMZ *in vivo*. U87 cells were injected subcutaneously into the flank of nude mice, and once tumors grew to approximately 50-75 mm^3^, mice were randomly treated with KU0060648 (low dose group, 10 mg/kg; high dose group, 50 mg/kg), TMZ (10 mg/kg), or their combination. As shown in Figure 7A, KU0060648 dose-dependently inhibited the growth of U87 tumor. However, 10 mg/kg TMZ alone did not make tumor size regress. Especially, the co-administration of low (10 mg/kg) or high dose (50 mg/kg) of KU0060648 with TMZ resulted in a substantial tumor growth inhibition compared with each agent alone, respectively, confirming that the synergistic effect between KU0060648 and TMZ *in vivo*. We further address the long-term survival prolonging effect of synergistic KU0060648/TMZ treatment. As shown in Figure 7B, the survival rate of U87 tumor-beard nude mice treated with co-administrated KU0060648/TMZ was profoundly increased compared with each agent alone.

We next determined the effect of the combination of KU0060648 and TMZ on the regulation of downstream effectors of AKT signaling and malignant markers in tumorous tissues. IHC analysis revealed that KU0060648 or KU0060648/TMZ treatment effectively inhibited the expression of p-DNA-PKcs and p-AKT (Figure 7C). In addition, staining of γH2AX was surveyed. Consistent with our *in vitro* study, significant increases of γH2AX levels only emerged in comparison between KU0060648/TMZ and TMZ groups, while not in KU0060648 treatment alone. KU0060648 or KU0060648/TMZ treatment also significantly suppressed the expression of Ki-67 (the biomarker of proliferation), c-Myc, survivin, MMP-9 and VEGF and enhanced the expression of apoptotic promoting biomarker caspase-3 in U87 tumors (Figure 7C). Collectively, these results suggested that combination of KU0060648 and TMZ was more efficient in inhibition of the U87 tumors than either of the single agents.

## DISCUSSION

In this study, we reported that the p-DNA-PKcs (S2056) was significantly elevated in glioma compared with adjacent normal brain, and such a high level of phosphorylated DNA-PKcs tightly associated with malignancy of glioma, including clinical stage and the survival time of glioma patients, collectively demonstrating a pivotal role of DNA-PKcs in the progression of glioma. This study also investigated the role of DNA-PKcs in TMZ sensitivity in glioma cells. The results indicated that inhibition of DNA-PKcs activation decreased glioma cell malignancies and promoted TMZ efficacy. Specifically, we demonstrated that inhibitor of DNA-PKcs, namely the KU0060648, functioned mainly due to suppression of AKT signaling.

The most well-characterized factor that controls DNA-PKcs kinase activity is the broken ends of DNA double-strand breaks (DSBs), which is generated either endogenously or exogenously [29]. Binding to DNA initiates the activation of DNA-PKcs kinase. Once activated, DNA-PKcs kinase phosphorylates and alters the activity of proteins that mediate NHEJ involved in DNA damage response, including phosphorylating histone variant H2AX (γH2AX) at Ser139 either directly or indirectly through AKT/GSK3β signaling [30]. In order to see if the hyperactivated DNA-PKcs was derived from DSBs, we investigated the expression of γH2AX in paired glioma samples using IHC. Our data revealed a poor correlation between p-DNA-PKcs and γH2AX, suggesting that activation of DNA-PKcs in glioma was not necessarily dependent on DSBs. Recently, growing evidences have characterized that, in addition to DSBs, several molecules might also perform as key modulators of DNA-PKcs activity in human cells. For example, Liccardi G et al. reported that nuclear translocated EGFR could bind to DNA-PKcs and enhance DNA-PKcs activity [31]. Besides, Olsen BB et al. showed a critical role of protein kinase CK2 in DNA-PKcs activation in glioblastoma cells [32]. Combined with these studies, our data that the upregulated p-DNA-PKcs levels in glioma samples did not correlate with DSB load indicated multiple underlying mechanisms might account for activation of DNA-PKcs in various tumors.

DNA-PKcs plays a great part in classic DNA damage repair pathway. Inhibition of DNA-PKcs is therefore an attractive approach to modulating resistance to therapeutically induced DNA insults. Current cancer therapy invariably utilizes a combination of chemotherapeutic agents and numerous reports have documented the *in vitro* antiproliferative effect of DNA-PKcs inhibitors [33 - 35]. NU7026 and NU7441, two DNA-PKcs inhibitors, have been clarified to improve the efficacy of various chemotherapeutic drugs, such as etoposide, cisplatin and doxorubicin in some types of solid tumors, including colon cancer, non-small cell lung cancer (NSCLC), leukemia or HCC [36 - 38]. We described here that inhibition of DNA-PKcs by newly-designed inhibitor KU0060648 dose-dependently enhanced the cytotoxic effect of TMZ in glioma cells. Furthermore, the combination of KU0060648 and TMZ could also increase the apoptotic rates. Notably, KU0060648 alone did not enhance the DNA damage in glioma cell lines, suggesting that inhibition of DNA damage repair might not account for, at least not predominant, mechanism that KU0060648 suppressed glioma malignancies.

Poor prognosis and high lethality of glioma is largely attributed to the high invasion and angiogenesis property of glioma cells [39, 40], which is clinically linked to chemotherapy resistance [41, 42]. These malignant phenotypes essentially support glioma cell proliferation and spreading. Our data demonstrates that KU0060648 effectively inhibited the glioma invasion and angiogenesis *in vitro*. However, we noticed that administration of 100 μM TMZ slightly promoted these malignant properties of glioma. Such a phenomenon of “chemotherapy induced tumor invasion and angiogenesis” is not a new story in the preclinical literatures, but is often overlooked as a mechanism that may contribute to eventual resistance and disease progression following therapy failure [43, 44]. Several studies have revealed that activation of DNA-PKcs might be a key tone to facilitate cancer cell invasion and angiogenesis, through coordinately regulating the expressions of related effectors or activities of signals [45, 46]. In our study, we discovered that TMZ treatment alone induced the activation of DNA-PKcs, then upregulated the level of downstream effectors. When KU0060648 was added, those adverse effects of TMZ were eliminated and even brought about a synergistic inhibition of glioma invasion and angiogenesis.

The synergistic effect of KU0060648 and TMZ was also observed in the treatment of glioma *in vivo*. High dose of KU0060648 addition to TMZ had a more remarkable anti-tumor effect than the low dose of KU0060648 in the presence of TMZ as well as KU0060648 at its high dose alone, further suggesting the feasibility of combination of DNA-PKcs inhibitor and TMZ on the glioma growth. 100 μM TMZ alone could not upregulate the survival time in animal harboring U87 tumors. However, with increased dose, KU0060648 sufficiently extended the survival time of TMZ treatment group, confirming that the combination of KU0060648 and TMZ had a synergistic effect for prolonging the survival period of nude mice harboring U87 xenograft tumors. Collectively, these data expanded the feasibility of the combination of the DNA-PKcs inhibitor with chemotherapeutic agents in solid tumor treatment.

The present study implies that the molecular mechanism underlying DNA-PKcs inhibition-enhanced TMZ efficacy can be majorly related to the inhibition of AKT signaling. Constitutive activation of AKT exists in a variety of malignances including glioma, and is closely correlated with cancer development and progression [47, 48]. Specifically, hyperactivation of AKT also confers cell resistance to many chemotherapy agents [49, 50]. As we found, allosteric AKT inhibitor MK2206 mimicked the effect of KU0060648 that substantially decreased cell growth and sensitized TMZ efficacy, suggesting KU0060648 functioned mainly through suppression of AKT. Further investigations confirmed the inhibitory effect of KU0060648 on DNA-PKcs/AKT signal. More importantly, we disclosed that KU0060648 induced inhibition of AKT activation may be, at least partly, derived from its disruption of the interaction between DNA-PKcs and AKT in glioma cells. Consistently, the downstream molecules of AKT, such as c-Myc, and AKT-related anti-apoptotic, metastatic or angiogenic molecules could be efficiently suppressed by KU0060648 treatment. Many studies have shown that these substrates participate in the resistance of certain cancers to chemotherapy [51 - 55]. Taken together, these data revealed that the single agent of KU0060648 or in combination with TMZ in glioma treatment mainly depended on inhibition of AKT signaling.

A series of work from Brian Hemmings groups has identified DNA-PKcs, as a central node, amplifies and conveys signals from the damaged DNA to DSBs repair and anti-apoptosis machineries, for example through AKT pathway, thus promoting survival [56, 57]. However, role of activated DNA-PKcs/AKT axis without chemo- or radiotherapy triggered has been completely ignored, which is certainly existed as our result illustrated. In fact, mountains of researches have observed that DNA-PKcs promotes tumor malignancies through stimulating and integrating an extensive network of cellular signaling which is not involving DNA damage repair [20, 23, 46]. Actually, *in vitro* assay has demonstrated that synthetic peptides of DNA-PKcs can phosphorylate AKT on Ser 473 [58], which provided a fundament that activated DNA-PKcs, whatever DSBs induced or non-DSBs induced, could potentially play an additional role in enhanced AKT signaling. This was confirmed by our clinical investigation that a linear correlation between p-DNA-PKcs and p-AKT expression was discovered. Such a finding shed light on the overlooked function of non-DSBs stimulated DNA-PKcs/AKT signal, which potentially promotes glioma progression.

In conclusion, our findings suggest that overactivation of DNA-PKcs is clinically and functionally relevant to the progression of human glioma, and mediates TMZ resistance in glioma treatment. Disrupting the DNA-PKcs/AKT interaction and consequently regulating the downstream effectors of AKT may provide a potential mechanism by which suppression of DNA-PKcs activity can sensitize the response to TMZ and possibly several other chemotherapeutic agents.

## MATERIALS AND METHODS

### Cell lines and cell culture

The human glioma cell lines U373, A172, M059K were obtained from the American Type Culture Collection (ATCC, Manassas, VA, USA); H4, U87, U251 were purchased from the Cell Culture Center (Chinese Academy of Medical Sciences, Beijing, China). All these cell lines were maintained in Dulbecco's modified Eagle's medium (DMEM; Invitrogen, Carlsbad, CA, USA) supplemented with 10% fetal bovine serum (FBS; HyClone, Logan, UT, USA) and at 37°C in 5% CO_2_.

### Chemical agents and antibodies

KU0060648, Temozolomide, MK-2206, PD98059 were obtained from Merck Millipore. Anti-total DNA-PKcs, anti-phospho-DNA-PKcs (p-DNA-PKcs, Ser 2056) and anti-VEGF antibodies were purchased from Abcam. Anti-AKT, anti-p-AKT (Ser 473), anti-p-histone H2AX (γH2AX, Ser 139), anti-GSK3β, anti-p-GSK3β (Ser 9), anti-survivin, anti-Mcl-1, anti-c-Myc, anti-MMP9, anti-Ki-67, anti-cleaved caspase-3 antibodies, anti-Actin, anti-mouse and anti-rabbit secondary antibodies were purchased from Cell Signaling Technology.

### Patient information and tissue specimens

In total, 217 paraffin-embedded, archived glioma samples and their respective adjacent non-cancerous tissues were obtained from the Sanbo Brain Hospital, Capital Medical University (Beijing, China). Fresh gliomas and adjacent non-tumor tissues were collected using protocols approved by the Ethics Committee of Sanbo Brain Hospital, and informed consent was obtained from all patients. Normal brain tissues (2 fresh and 21 paraffin-embedded samples) were obtained from car accident patients who received craniotomy in our hospital, and had been ethnically approved for scientific applications as mentioned before [59]. The clinical and pathological classification and stage were determined according to the WHO classification of brain tumors criteria. The clinical information for the patient samples is summarized in Supplementary Tables S1 and S2 (see Supplementary material).

### Immunohistochemistry (IHC)

Briefly, tissue sections were deparaffinized, soaked in Tris-EDTA buffer (pH 8.0) and boiled in the microwave, then incubated with the primary antibodies at 4°C overnight. Next day, slides were washed and stained by the secondary antibody and DAB disclosure, counterstained with hematoxylin, dehydrated and mounted. The sections were reviewed and scored independently by two observers. Degree of immunostaining was determined based on both the proportion of positively stained tumor cells and the intensity of staining. The proportion of positive tumor cells was scored as follows: 0, no positive tumor cells; 1, < 10%; 2, 10%–35%; 3, 35%–75%; 4, > 75%. The intensity of staining was graded according to the following criteria: 1, weak staining (light yellow); 2, moderate staining (yellow–brown); 3, strong staining (brown). The IHC score was calculated as staining intensity score × proportion of positive tumor cells. Using this method of assessment, the expression of p-DNA-PKcs was scored as 0, 1, 2, 3, 4, 6, 8, 9, and 12. High expression of p-DNA-PKcs and p-AKT referred to IHC score ≥ 6 and low expression of p-DNA-PKcs and p-AKT was defined as IHC score < 6.

### Short interfering RNA transfection (siRNA)

Specific siRNA targeting DNA-PKcs was designed and provided by Ribobio (Cat#: siG0812181317001 and siG0812181317002). Transfection of siRNA was done according to the manufacturer's protocol. Briefly, cells were plated in 60-mm culture plates at 5×10^5^ cells per well, grown for 12 h, then transfected with siRNA using Lipofectamine 2000 (Invitrogen). Transfected cells were incubated at 37°C with 5% CO_2_ for 24 h.

### Cell proliferation/viability

Proliferation/viability of cells was determined using MTS assay as previously described with minor modifications [60]. Briefly, a total of 3 × 10^3^ cells in 100 μL of 10% FBS culture medium were seeded in 96-well plates. Once confluent, cells were cultured for 72 h before analysis. Then, the medium was aspirated and incubated with MTS solution (Promega, Madison, WI, USA) for 1 h. The viable cell number was reflected as the MTS absorbance which was measured spectrophotometrically at 490 nm. For evaluating the long-term proliferation of cells (colony formation assay), 1 × 10^3^ tumor cells were plated into 60-mm dishes in 10% FBS culture medium. After 14 days, the cells were washed with PBS, fixed with methanol and 0.1% crystal violet. The colonies were counted and then photographed. All experiments were carried out in triplicate.

### AnnexinV/PI flowcytometry analysis

The Annexin V-FITC early apoptosis detection kit (Neobioscience, Shenzhen, China) was used to identify the apoptotic cells. Briefly, approximate 10^5^ cells were harvested, washed with cold PBS twice and resuspended with 350 μL 1 × Binding Buffer. Then, 5 μL of the Annexin V-FITC conjugate was added. After 20 minutes' light-prevented incubation at room temperature, cell suspension was diluted to a final volume of 500 μL/assay with ice cold 1 × Binding Buffer. Next, 10 μL of the Propidium Iodide (PI) solution were added to each sample tube, and the samples were analyzed by FACS Canto™II cell analyzer (BD Biosciences, San Jose, CA, USA).

### TUNEL assay

As previously described [61], One-Step TUNEL Apoptosis Assay Kit (Beyotime, Jiangsu, China) was applied in TUNEL assay according to the manuscript. Nuclei were stained with 4′, 6-diamidino-2-phenylindole (DAPI). Staining was evaluated using fluorescence microscopy.

### Hoechst 33258 staining

Hoechst 33258 staining was performed as described previously with minor modifications [62]. Briefly, cells were fixed with 4% formaldehyde solution for 30 min at room temperature and washed twice with PBS. Fixed cells were stained with Hoechst 33258 of 50 ng/mL and incubated for 30 min at room temperature and washed with PBS. Apoptotic cells were identified by condensation and fragmentation of nuclei examined by an Olympus IX71 fluorescence microscope. The apoptotic rate of cell population was calculated as the ratio of apoptotic cells to total cells counted ×100. A minimum of 500 cells were counted for each treatment.

### Caspase 3/7 activity assay

After cells being treated with reagents either alone or in a combination for 48 hours, the activity of caspase-3/7 was evaluated using Caspase-Glo 3/7 Assay Kit (Promega Corporation) according to manufacturer's instruction. Results were expressed as relative luminescence units (RLU).

### γH2AX foci assay

Cells were grown on coverslips at a density of 1×10^5^ cells per well. After treatment with TMZ (100 μM) or additional KU0060648 (1 μM and 10 μM) for distinct time periods, cells were fixed in 4% paraformaldehyde for 10 min at room temperature and incubated in 0.1% Triton X-100 for 20 min, then blocked with 5% normal goat serum (Sigma-Aldrich). The cells were reacted with anti-γH2AX (1:500) at 4°C overnight. After that, cells were incubated with FITC- (fluorescein isothiocyanate) conjugated secondary antibodies for 1 h at 37°C. Nuclei were stained with DAPI (4′,6-diamidino-2-phenylindole) at a final concentration of 0.1 μg/mL. Images were visualized and recorded with a Zeiss LSM780 confocal microscope (Carl Zeiss Inc., Oberkochen, Germany).

### Transwell invasion assay

The transwell invasion assay was performed using the transwell chamber with a Matrigel-coated filter. A total of 5 × 10^4^ cells to be tested were starved in serum and growth factor-free medium overnight and then plated on the top chamber with or without agents as indicated for 18 h, followed by removal of cells inside the upper chamber with cotton swabs, and the invasive cells on the lower side were fixed, stained with 0.1% crystal violet solution and counted using light microscope. The experiment was repeated three times.

### HUVEC tube formation assay

Matrigel (50 μL) was pipetted into each well of a 12-well plate and polymerized for 2 hours at 37°C. HUVECs (1 × 10^4^) in 150 μL of conditioned medium from each treatment group were added to each well and incubated at 37°C in 5% CO_2_ for 24 h. Pictures were taken under a × 100 bright-field microscope. The experiment was repeated three times.

### Real-time PCR (RT–PCR)

Total RNA from cells was extracted with TRIzol (Invitrogen). First-strand cDNA was synthesized by using the Superscript II-reverse transcriptase kit (Invitrogen) according to the manufacturer's instructions. Real-time PCR (qPCR) was conducted using SYBR Premix Ex Taq (Takara) on an ABI 7300 Real-Time PCR System (Applied Biosystems). All samples were normalized to GAPDH. Gene-specific qPCR primer pairs are provided in as below.

**Table:** 

	Forward primer	Reverse primer
**Survivin**	CCACCGCATCTCTACATTCA	TATGTTCCTCTATGGGGTCG
**Mcl-1**	CTTACGACGGGTTGGG	GGTTCGATGCAGCTTTCTTGG
**c-Myc**	TGACGAGACCTTCGTGAAGA	ATTGATGTTATTTACACTTAAGGGT
**MMP-9**	GCCTGGCACATAGTAGGCCC	CTTCCTAGCCAGCCGGCATC
**VEGF**	ATCTTCAAGCCATCCTGTGTGC	GCTCACCGCCTCGGCTTGT
**GAPDH**	ACAGTCAGCCGCATCTTCTT	GACAAGCTTCCCGTTCTCAG

### Immunoblotting

Total cell protein extracts were separated on 10% or 15% SDS–PAGE and transferred onto polyvinylidene fluoride (PVDF) membranes. The membranes were subsequently probed with indicated primary antibodies and anti-mouse or anti-rabbit secondary antibodies, respectively. All of the first antibodies were diluted at 1:1000 except for Actin at 1:5000. The chemiluminescence signal was detected with Luminescent Image Analyzer LAS-4000 (Fujifilm). Blotting membranes were stripped and reprobed with anti-Actin as a loading control.

### Immunoprecipitation (IP)

For immunoprecipitation assays, cells were washed with cold PBS and lysed with cold lysis buffer (50mM Tris-Cl, pH 7.4, 150mm NaCl, 1mm EDTA, 1% NP-40, 0.25% sodium deoxycholate and protease inhibitor mixture) at 4°C for 30 min. Cellular extracts were incubated with appropriate primary antibodies on rotator at 4°C overnight, followed by the addition of protein A/G sepharose beads for 2 h at 4°C. Beads were then washed five times with lysis buffer. The immune complexes were subjected to SDS–PAGE followed by IB with secondary antibodies.

### Xenograft studies

Female, 5 weeks old, Nu/Nu mice were purchased from Vital River laboratories (Beijing, China). All animal care and experiments were carried out according to the Institutional Animal Welfare Guidelines of Chinese Academy of Medical Sciences. A total of 1×10^6^ U87 cells were injected subcutaneously into mice. To assess tumor growth, treatment began 2 weeks after injection of tumor cells. Mice were randomly divided into 6 groups (n = 5 per group): Control group: normal saline intraperitoneal (i.p.) injection once day; single-agent KU0060648 group: 10 mg/kg (low dosage) or 50 mg/kg (high dosage) KU0060648 i.p. once daily for 30 days; TMZ group: 10 mg/kg i.p. once daily for 30 days, and KU0060648/TMZ combination group: KU0060648 was administered i.p. once daily for 5 days with the first dose immediately before 10 mg/kg TMZ. At the end of each experiment, animals were sacrificed, and tumors were calculated and paraffin-embedded. Sections of 5.0 μm were cut and subjected to IHC staining.

### Statistical analysis

Statistical analyses were performed using SPSS 17.0 software (SPSS Inc., Chicago, IL, USA) and GraphPad Prism 5.0 (GraphPad software Inc., La Jolla, CA, USA). Survival curves were plotted using Kaplan–Meier estimates. Wilcoxon rank sum test was used for statistical analysis of clinical scores; χ^2^ (chi-square) test was applied in studying the correlation between γH2AX and p-DNA-PKcs, and p-DNA-PKcs and p-AKT. Comparisons between 2 groups were performed using the Student's *t* test. Bivariate correlations between study variables were calculated by Pearson's correlation coefficients. One-way ANOVA test was used for statistical analysis of remaining data. All tests were two-tailed Data are presented as means ± SD. P < 0.05 was considered statistically significant.

## SUPPLEMENTARY TABLES AND FIGURES


