# Supplementary material for: Targeting hyperactivated DNA-PKcs by KU0060648 inhibits glioma progression and enhances temozolomide therapy via suppression of AKT signaling
Source: Oncotarget. 2016 Jul 27;7(34):55555–71. doi: 10.18632/oncotarget.10864 (PMC5342436; doi:10.18632/oncotarget.10864)
Supplement: Supplementary file 1 [file oncotarget-07-55555-s001.pdf]

Targeting hyperactivated DNA-PKcs by KU0060648 inhibits glioma progression and enhances temozolomide therapy via suppression of AKT signaling

SUPPLEMENTAL TABLES AND FIGURES

Supplementary Table S1: Overexpression of p-DNA-PKcs in human gliomas

	Numbers	p-DNA-PKcs(S2056) Status no.(%)		*P* Value
		Low (IHC<6)	High (IHC≥6)	
Adjacent non-tumor tissues	217	155(71.4%)	62(28.6%)	<0.0001
Glioma tissues	217	93(42.8)	124(57.2%)	

Supplementary Table S2: Multivariate analysis of p-DNA-PKcs and other prognostic factors

Categorical Variables	Overall survival Exp(B) for 95% CI (Lower and Upper)	*P* Value
Debulking Degrees (Complete resection vs. partial resection)	0.878 (0.628–1.228)	0.449
Age (< 45vs. >45)	0.885 (0.633–1.237)	0.474
Gender (Male vs. Female)	0.906 (0.657–1.248)	0.613
Tumor Locations (supratentorial vs subtentorial)	0.997 (0.721–1.377)	0.984
Tumor Grade (WHO calssification)	2.894 (2.114 – 3.960)	<0.001
P-DNA-PKcs(S2056) (High vs. Low)	3.052 (2.204–4.572)	<0.001

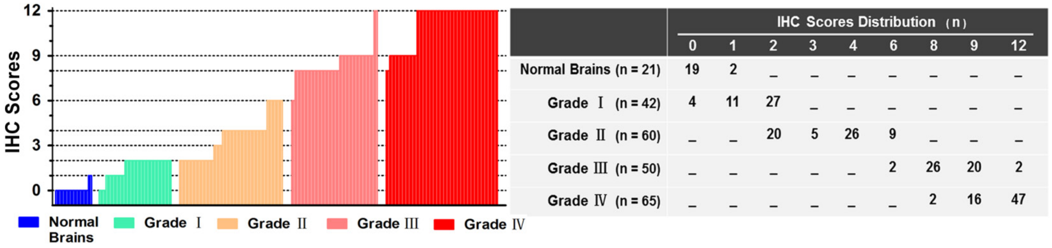

Supplementary Figure S1: Quantified IHC analysis of p-DNA-PKcs expression in normal brain tissues and human gliomas. Left: IHC scores; Right: detail information.

A

	IHC Scores Distribution (n)								
	0	1	2	3	4	6	8	9	12
Grade I (n = 25)	5	3	_	_	6	4	4	2	1
Grade II (n = 45)	11	3	4	2	1	15	4	3	2
Grade III (n = 32)	7	_	2	_	_	15	_	7	1
Grade IV (n = 53)	8	9	4	2	11	4	3	5	7

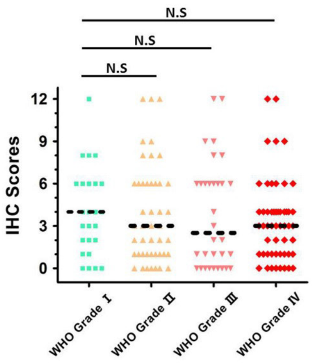

B

γH2AX Status NO.(%)	p-DNA-PKcs(S2056) Status NO.(%)	
	Low (IHC<6)	High (IHC≥6)
Low (IHC<6)	35 (22.6%)	43 (27.7%)
High (IHC≥6)	26 (16.8%)	51 (32.9%)

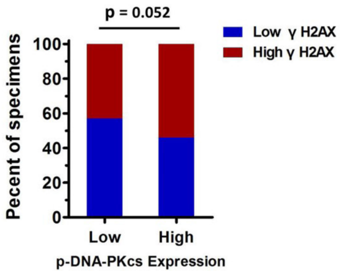

Supplementary Figure S2: IHC study on γH2AX expression in different grades of gliomas and correlation analysis between γH2AX and p-DNA-PKcs. A cohort of 155 patients with primary glioma occurrence and null chemo- or radiotherapy before surgery was founded. **A.** Left: global view of IHC staining; Right: correlation between γH2AX expression and tumor grade in surveyed cohort. (Bars, median expression values of IHC scores; N.S, no significance; Wilcoxon rank sum test); **B.** Correlation between γH2AX and p-DNA-PKcs (chi-square test).

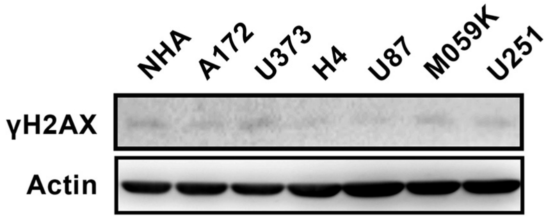

Supplementary Figure S3: Expression of γH2AX in NHA and glioma cell lines.

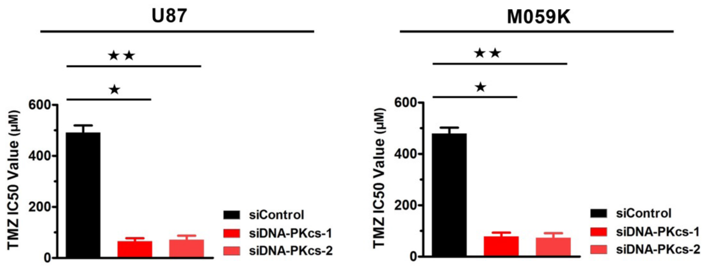

Supplementary Figure S4: Depletion of DNA-PKcs expression sensitizes glioma to TMZ in vitro. TMZ IC_{50} in U87 and M059K measured after 48 h of treatment with a non-targeting siRNA control or two specific DNA-PKcs siRNAs (Bars, SD; ★★, p <0.001, one-way ANOVA test).

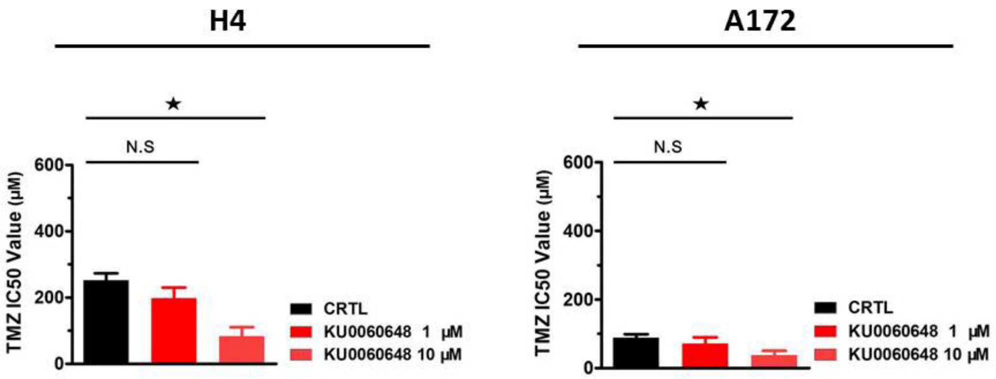

Supplementary Figure S5: (D) TMZ IC_{50} of H4 and A172 in the absence or presence of KU0060648. Two cell lines were incubated for 3 days in a range of concentrations of TMZ with or without of 1 or 10 µM KU0060648 and IC_{50} value was then calculated by MTS assay (Bars, SD; ★, p < 0.05; N.S, no significance; one-way ANOVA test).

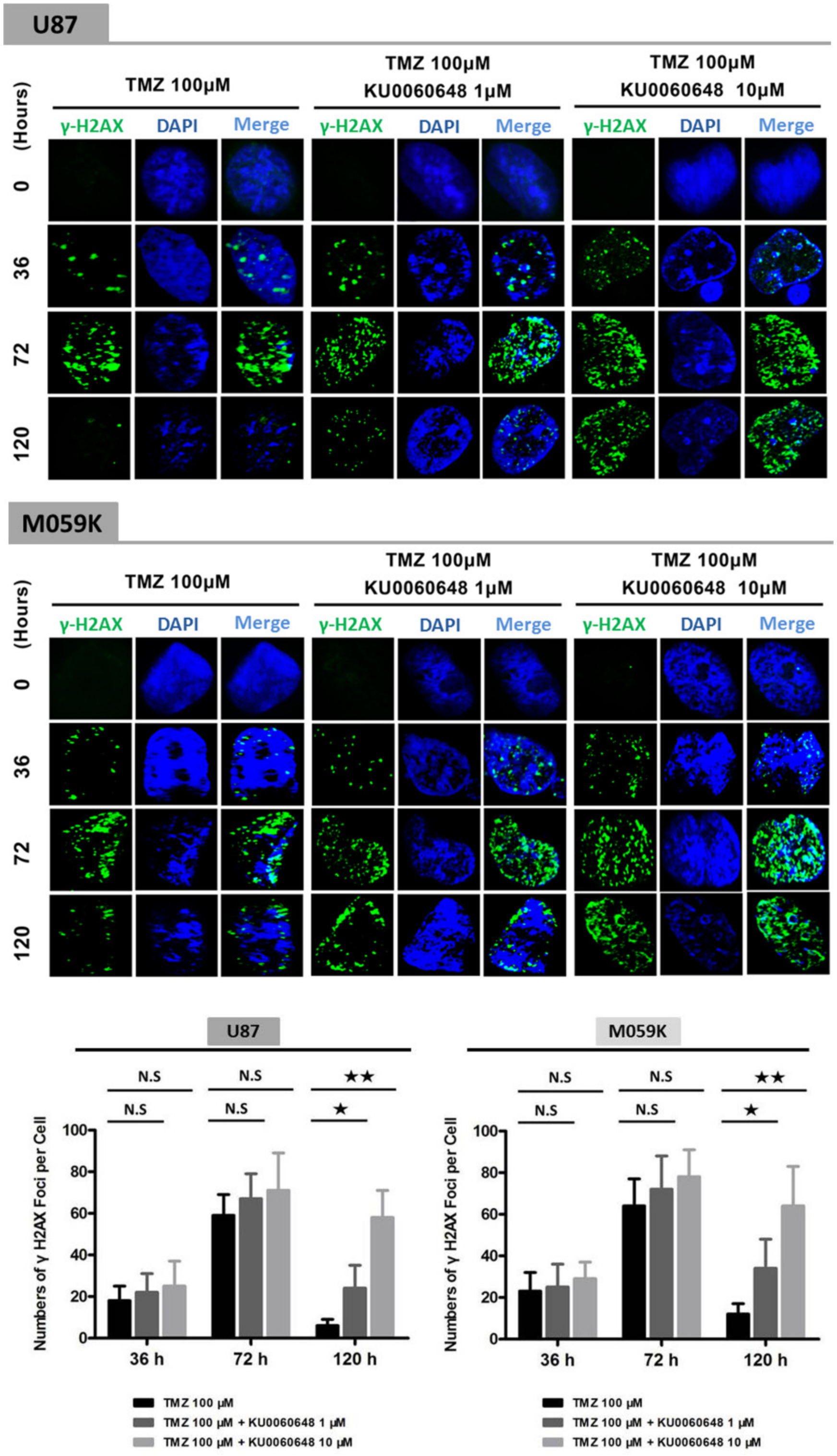

Supplementary Figure S6: γH2AX foci analysis in combined treatment of TMZ and KU0060648. Representative images of γH2AX foci in U87 and M059K cells (up) demonstrated that KU0060648 did not affect the level of TMZ-induced foci formation, but significantly retarded the loss of γH2AX foci (down) (Bars, SD; N.S, no significance; ★, $p < 0.05$; ★★, $p < 0.001$, one-way ANOVA test).

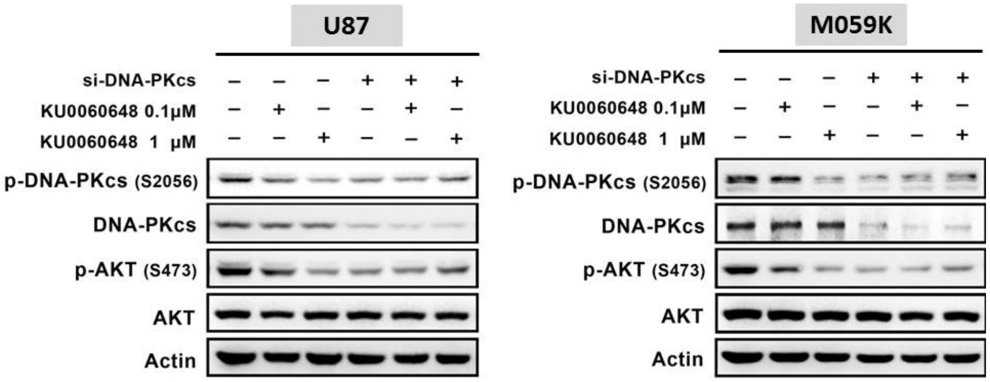

Supplementary Figure S7: Depletion of DNA-PKcs eliminates KU0060648 regulated- inhibition of DNA-PKcs/AKT axis. Immunoblot analysis of U87 and M059K which were incubated with KU0060648 (0.1 μM or 1 μM) 6 h after treatment of a non-targeting siRNA control or two specific DNA-PKcs siRNAs.
